# Rectus sheath haematoma or leaking aortic aneurysm - a diagnostic challenge: a case report

**DOI:** 10.1186/1752-1947-3-97

**Published:** 2009-11-03

**Authors:** Aidan G Shaw, Simon Fleming, Polly Drew, Jonathan N Lund, Manjeet Riyat

**Affiliations:** 1 School of Graduate Medicine and Health, University of Nottingham, Derby, DE22 3DT, UK; 2 Department of Surgery, Derby City General Hospital, Derby, DE22 3NE, UK; 3 Department of Emergency Medicine, Derbyshire Royal Infirmary, Derby, DE1 2QY, UK

## Abstract

**Introduction:**

A rectus sheath haematoma is a rare condition that arises from the accumulation of blood within the rectus sheath from either muscular tear or rupture of the epigastric vessels. It is a known complication of either blunt abdominal trauma, anticoagulation therapy or the repetitive contraction of the rectus muscle such as paroxysms of coughing. It remains an uncommon and elusive entity and is often clinically misdiagnosed.

**Case presentation:**

An 80-year-old British man with a known aortic aneurysm was admitted with sudden onset of right iliac fossa pain. The patient was hemodynamically stable and underwent a computed tomography scan which revealed an intact aorta and an acute rectus sheath hematoma.

**Conclusion:**

To the best of our knowledge, no case has previously been reported involving the diagnostic challenge of a rectus sheath hematoma in a patient with a known aortic aneurysm. Here we discuss the symptoms and signs of a rectus sheath hematoma, as well as the radiological modalities that could be utilized to reach the diagnosis.

## Introduction

A rectus sheath haematoma is a rare condition that arises from the accumulation of blood within the rectus sheath from either muscular tear or rupture of the epigastric vessels. It is a known complication of either blunt abdominal trauma, anticoagulation therapy [1,2] or the repetitive contraction of the rectus muscle such as paroxysms of coughing [3]. Other uncommon conditions associated include exacerbation of asthma [4], pregnancy [5], insulin injections [6], recent surgery and increased age [3]. It remains an uncommon and elusive entity and is often clinically misdiagnosed. To the best of our knowledge, no case has previously been reported involving the diagnostic challenge of a rectus sheath hematoma in a patient with a known aortic aneurysm. Here we discuss the symptoms and signs of a rectus sheath hematoma, as well as the radiological modalities that could be utilized to reach the diagnosis.

## Case presentation

An 80-year-old British Caucasian man with a known abdominal aortic aneurysm was brought to the Accident and Emergency department in acute abdominal pain. His last ultrasound scan had measured the aneurysm at 5.8 cm and he was currently awaiting endovascular repair.

The pain was located in the right iliac fossa, was of sudden onset, and was exacerbated by coughing and movement. He was, at that time, recovering from a chest infection and this had led to a non-productive cough for a week. There was no recent history of trauma.

He had a past medical history of myocardial infarction, coronary artery bypass graft and a perforated duodenal ulcer. He was receiving only antihypertensive therapy and was not taking any anticoagulation medication.

During examination, he was alert and in considerable discomfort. His pulse rate was 74/minute, his blood pressure was 147/81 mmHg, and his respiratory rate 16/minute. Oxygen saturations were 98% on air. Abdominal examination revealed a soft abdomen with a prominent mid-line laparotomy scar. Tenderness to palpation was present in the right iliac fossa, as well as guarding in this area. Blood tests revealed a normocytic normochromic anaemia (9 g/dl), a mild leucocytosis (13 × 10^9^/l) and a normal clotting profile (INR <1.0, platelets 232 × 10^9^/l).

**Figure 1:**
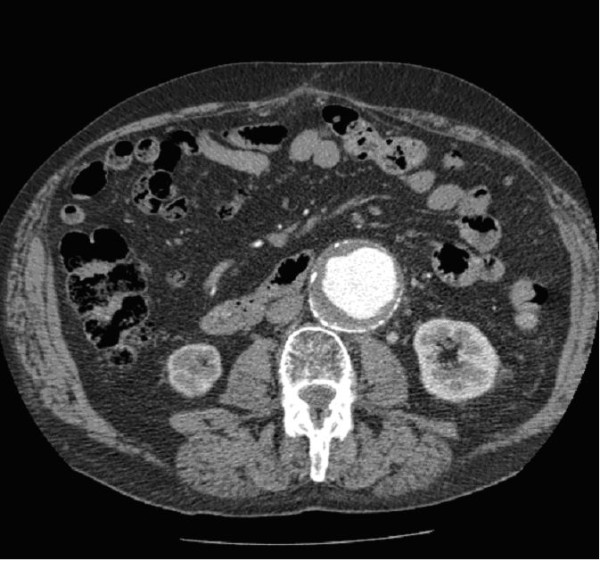
**Computed tomography scan showing a densely calcified aorta measuring 5.9 cm**.

**Figure 2:**
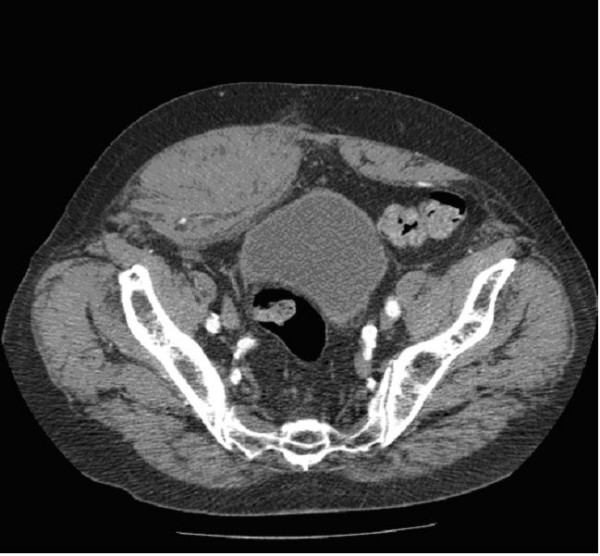
**Computed tomography showing a right inferior rectus sheath hematoma**.

A leaking abdominal aortic aneurysm was the first concern and the appropriate management followed. Large bore access was obtained and intravenous fluid therapy commenced. An urgent referral was made to the general surgeons who reviewed the patient in the emergency department. Laparotomy was discussed but as the patient was hemodynamically stable it was decided that contrast computerised tomography (CT) should be performed first. This demonstrated a densely calcified infrarenal aortic aneurysm measuring 5.8 cm with no evidence of a leak, and an acute right inferior rectus sheath haematoma (Figures 1 and 2).

The patient was admitted to the Surgical Admissions Unit where a line of conservative management was followed and he was subsequently discharged a few days later.

## Discussion

A rectus sheath haematoma in the initial stages can mimic an acute abdomen, including a ruptured aortic aneurysm [7], and a number of patients have received an exploratory laparotomy on the account of difficulty in differentiating between this diagnosis and intra-abdominal pathology [8]. It is thought that with careful history taking and attention to a few critical points on examination, one could reliably differentiate a rectus hematoma from intra-abdominal pathology [9] thereby avoiding unnecessary surgery.

In 1926, Carnett first described a technique to differentiate between intra-abdominal pathology and pathology of the abdominal wall [10]. The patient is first laid supine and the point of maximal tenderness is identified. Carnett's test is said to be positive when the patient is then asked to sit up halfway and experiences increased discomfort on palpation of the tender area. The implication here is that the pathology is located in the abdominal wall. If the pain were alleviated upon sitting then the pathology would be intra-abdominal, as the contraction of the rectus muscle protects the viscera. Thomson and Francis assessed this test in 120 patients and found it to be a reliable means of identifying abdominal wall pathology, saving the expense and risks associated with investigation, and even the risk of unnecessary surgery [11].

Diagnosis of a rectus sheath hematoma is reached by combining medical history, examination and radiological investigation, which can be performed through ultrasonography or CT.

Ultrasonography is usually the first radiological investigation of choice. In the presence of an aortic aneurysm, a FAST (Focused Assessment with Sonography for Trauma) scan can be performed as a first-line investigation. However, in this case, a FAST scan would only have confirmed that there was no intraperitoneal fluid and unless the operator had clinical awareness of the possible differential diagnosis, a rectus sheath hematoma certainly would have been missed.

CT is considered a more sensitive investigation than ultrasonography. Gallego *et al. *compared ultrasonography with computed tomography for accuracy of diagnosis and avoidance of surgery for rectus sheath hematoma. They found that ultrasonography was diagnostically accurate in 71% of patients imaged, and CT was diagnostically accurate in 100% of cases [13].

## Conclusion

To the best of our knowledge, this is the first reported case of an acute rectus sheath hematoma with a concurrent abdominal aortic aneurysm. Even in a patient without known intra-abdominal pathology, a high index of clinical suspicion and correct radiological investigation is required to achieve the correct diagnosis. In our patient's case, an aneurysm rupture will always remain the diagnosis of exclusion and in the presence of a hemodynamically stable patient, a CT scan would differentiate between the two diagnoses negating the need for emergency surgery.

## Abbreviations

CT: computer tomography; FAST: Focused Assessment with Sonography for Trauma.

## Consent

Written informed consent was obtained from the patient for publication of this case report and any accompanying images. A copy of the written consent is available for review by the Editor-in-Chief of this journal.

## Competing interests

The authors declare that they have no competing interests.

## Authors' contributions

AS wrote the manuscript; SF contributed to the manuscript. SF, JL and MR reviewed the literature. All authors contributed intellectual content, have read and approved the final manuscript
